# Sensitive and Facile Detection of Aloin via N,F-CD-Coated Test Strips Coupled with a Miniaturized Fluorimeter

**DOI:** 10.3390/biom15071052

**Published:** 2025-07-21

**Authors:** Guo Wei, Chuanliang Wang, Rui Wang, Peng Zhang, Xuhui Geng, Jinhua Li, Abbas Ostovan, Lingxin Chen, Zhihua Song

**Affiliations:** 1 School of Pharmacy, Collaborative Innovation Center of Advanced Drug Delivery System and Biotech Drugs in Universities of Shandong, Key Laboratory of Molecular Pharmacology and Drug Evaluation (Yantai University), Ministry of Education, Yantai University, Yantai 264005, China; w13573156391@126.com (G.W.); 17606453912@163.com (R.W.); zp19877891@126.com (P.Z.); 2 CAS Key Laboratory of Separation Sciences for Analytical Chemistry, Dalian Institute of Chemical Physics, Chinese Academy of Sciences, 457 Zhongshan Road, Dalian 116023, China; wangcl@dicp.ac.cn (C.W.); xhgeng@dicp.ac.cn (X.G.); 3 University of Chinese Academy of Sciences, Beijing 100049, China; 4 CAS Key Laboratory of Coastal Environmental Processes and Ecological Remediation, Research Center for Coastal Environmental Engineering and Technology, Yantai Institute of Coastal Zone Research, Chinese Academy of Sciences, Yantai 264003, China; jhli@yic.ac.cn (J.L.); saman.ostovan@yahoo.com (A.O.); lxchen@yic.ac.cn (L.C.)

**Keywords:** aloin, nitrogen and fluorine-doped carbon dots (N,F-CDs), test strips, fluorescence analysis, real samples

## Abstract

Aloin, a kind of active phenolic component, is sourced from *Aloe vera*. Recently, the determination of aloin has received enormous attention, owing to its positive performance (including anti-tumor, antibacterial, detoxification, liver protection, anti-stomach damage, and skin protection activities) and painful side effects (increased carcinogenicity caused by excessive use of aloin) impacting human health. This investigation was inspired by the good fluorescence properties of carbon dots (CDs); CD-based sensors have aroused a great deal of interest due to their excellent sensitivity and selectivity. Thus, it is of great significance to develop novel CD-based sensors for aloin determination. Herein, N,F-CDs were designed and synthesized through a convenient hydrothermal strategy; the synthesized N,F-CDs possessed good fluorescence performance and a small particle size (near 4.3 nm), which demonstrated the successful preparation of N,F-CDs. The resulting N,F-CDs possessed a large Stokes shift and could emit a highly stable green fluorescence. The fluorescence of the N,F-CDs could be effectively quenched by aloin through the inner filter effect. Furthermore, the synthesis procedure was easy to operate. Finally, the N,F-CD-coated test strips were fabricated and combined with a miniaturized fluorimeter for the fluorescence detection of aloin via the inner filter effect for the first time. The N,F-CD-coated test strips were fabricated and used for the fluorescence sensing of aloin, and the results were compared with a typical ultraviolet (UV) method. The N,F-CD-coated test strips exhibited high recovery (96.9~106.1%) and sensitivity (31.8 nM, n = 3), good selectivity, low sample consumption (1 μL), high speed (5 min), good stability, and anti-interference properties. The results indicate that N,F-CD-coated test strips are applicable for the quantitative determination of aloin in bovine serum, orange juice, and urine samples.

## 1. Introduction

Aloin, a natural active product extracted from *Aloe vera*, contains multiple compounds involving polyhydroxy and benzene rings, etc. (Figure S1). Aloin has aroused a great deal of interest for many years, owing to its positive properties including anticancer, antiviral, antioxidant, antibacterial, liver protection, skin care, and anti-inflammatory effects [1,2,3,4]. Nevertheless, the excessive and/or improper utilization of aloin will cause painful side effects (such as carcinogenicity) impacting human health. Therefore, the aloin content of foods or beverages is strictly managed. For example, the European Union and the US Food and Drug Administration (FDA) have stipulated that aloin in foods and beverages should not exceed 0.1 mg/L and 10 mg/L, respectively [5]. Thus, it is very meaningful to develop many approaches for the quantitative detection of aloin in biosamples.

To date, diverse strategies, including quantitative nuclear magnetic resonance spectroscopy (qNMR) [6], the ultraviolet (UV) method [7,8], capillary electrophoresis [9], high-performance liquid chromatography (HPLC) [10], liquid chromatography–tandem mass spectrometry (LC-MS/MS) [11], and ultra performance liquid chromatography–quadrupole time-of-flight mass spectrometry (UPLC-Q-TOF-MS) [12], have been established for aloin determination. However, these frequently used techniques suffer from several limitations (such as being time-consuming and high-cost, and requiring skilled operators and sophisticated sample pretreatment processes). For example, a microwave-assisted extraction (MAE) procedure is very necessary in the LC-MS/MS method [13]. As a result, it is still critical to propose facile and convenient methods for aloin detection.

Carbon dots (CDs), a nanomaterial (possessing nanoscale graphitic or amorphous carbon cores and abundant functional groups, and being stabilized by oxidized surface groups), exhibit good performance in photosensitizers or light harvesters due to their excellent photostability, fascinating photophysical properties, low toxicity, low cost, and good solubility (in polar organic solvents or aqueous solutions) [14,15]. Recently, CD-based fluorescence analysis has aroused a great deal of interest in environmental monitoring and food safety, due to merits such as high sensitivity and selectivity, fast response, simplicity, low cost, and convenience [16,17,18]. Chen and Wang have fabricated highly fluorescent CDs (possessing a high-photoluminescence quantum yield of 71% and a narrow full width at half maximum of 65 nm) through a robust aluminum-based microfluidic chip platform [19]. Additionally, Jiang et al. synthesized CDs via a low-temperature microfluidic strategy (90 °C) for the highly sensitive detection of cefquinome (0.78 ng/mL) [20]. Currently, heteroatoms including oxygen (O), fluorine (F, possessing strong electronegativity), nitrogen (N), and sulfur (S), etc., have been doped into CDs, and the heteroatom-doped CDs have received sufficient attention due to their ability to reduce non-radiative recombinations and their remarkable fluorescence properties [21,22]. Kong’s research group fabricated N-CDs for the analysis of Fe^3+^ (1.0~80.0 μmol/L), and a high recovery rate resulted (98.5~109.2%) [23]. In addition, Bi and Xiao et al. synthesized F,N-CDs via a microwave-assisted heating method; the F,N-CDs exhibited photoactivated fluorescence enhancement, blue-shift in fluorescence emission (586 nm to 550 nm), and reversible piezochromic behavior; prominently, the F,N-CDs might be used as traditional pressure-sensitive materials, utilized for cellular imaging and optical recording systems [24]. Furthermore, F,N-CDs with thermally activated delayed fluorescence have been synthesized by Zhang and Yan’s research group [25].

**Figure 1:**
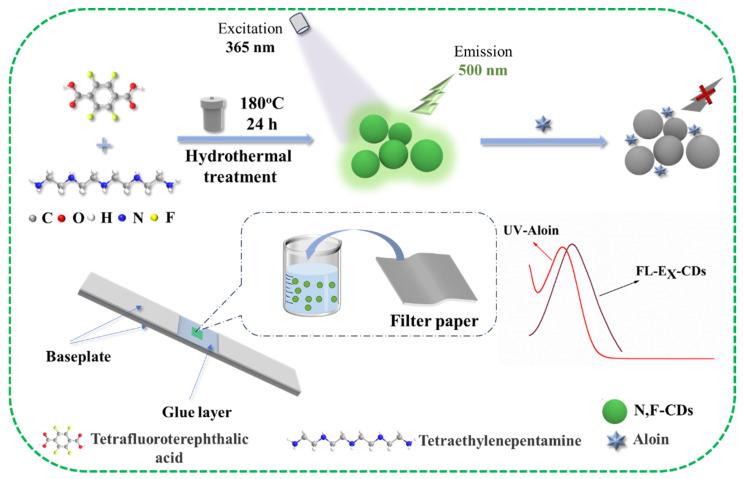
Schematic diagram of the preparation process of N,F-CDs and the detection principle of aloin.

It has been verified that the surface state and photoluminescence properties of CDs could be improved with N-doping and F-doping, which could further optimize the luminescence stability of CDs (owing to the largest electronegativity of F) [26]. Thus, it is crucial to develop novel strategies for the preparation of CDs and to investigate their applications. In this work, novel N- and F-doped carbon dots (N,F-CDs) were designed, synthesized, and used as a fluorescent probe to detect aloin, and the schematic diagram is illustrated in Figure 1. First, N,F-CDs were synthesized by the hydrothermal method using tetrafluoroterephthalic acid and tetraethylenepentamine as precursors. The N,F-CDs exhibited a green fluorescence under UV lamps, and their optimal excitation and emission wavelengths are 370 nm and 500 nm, with a Stokes shift of about 130 nm. In the N,F-CD-coated test strips, the fluorescence of the probe could be quenched by aloin. Moreover, the N,F-CD-coated test strips were constructed by immersing filter paper into the N,F-CD solution. Subsequently, the N,F-CD-coated test strips were coupled with a miniaturized fluorimeter for the quantitative determination of aloin in real samples.

## 2. Experimental Section

### 2.1. Materials and Reagents

Tetrafluoroterephthalic acid, tetraethylenepentamine, glycine, L-valine, and L-tryptophan were purchased from Shanghai Aladdin Biochemical Technology Co., Ltd. (Shanghai, China). Aloin (≥96%), L-proline, L-threonine, L-cysteine, L-glutamic acid, L-serine, and L-aspartic acid were purchased from Shanghai Macklin Biochemical Technology Co., Ltd. (Shanghai, China). Sodium hydroxide (NaOH) and hydrochloric acid (HCl) were supplied by Sinopharm Chemical Reagent Co., Ltd. (Shanghai, China). Sodium phosphate dibasic (NaH_2_PO_4_) was acquired from Sun Chemical Technology Co., Ltd. (Shanghai, China). Sodium phosphate monobasic dihydrate (NaH_2_PO_4_·2H_2_O) was purchased from Fuchen Chemical Reagents Co., Ltd. (Tianjin, China). Quantitative filter paper was supplied by Cytiva Bio-technology Co., Ltd. (Hangzhou, China). Fetal bovine serum was supplied by Zhejiang tian hang biotechnology Co., Ltd. (Zhejiang, China). Sodium chloride (NaCl), potassium chloride (KCl), and ammonium chloride (NH_4_Cl) were bought from Tianjin kermel chemical reagents Co., Ltd. (Tianjin, China), Tianjin beichen fangzheng chemical reagent Co., Ltd. (Tianjin, China), and Tianjin dengke chemical reagent Co., Ltd. (Tianjin, China), separately. Magnesium chloride (MgCl_2_) and sodium hydrogen bicarbonate (NaHCO_3_) were obtained from Tianjin berens biotechnology Co., Ltd. (Tianjin, China), and Tianjin hengxing chemical preparation Co., Ltd. (Tianjin, China), respectively. Purified water, used throughout this work, was supplied by Wahaha Group Co., Ltd. (Hangzhou, China).

### 2.2. Instruments

A miniaturized fluorimeter possessing excitation and emission wavelengths of 365 nm and 500 nm, was provided by a laboratory for micro-instruments for analytical chemistry (Dalian Institute of Chemical Physics, Chinese Academy of Sciences, Liaoning, China). And the data was recorded via the chromatographic workstation N2000 (Science Technology (Hangzhou) Inc., Hangzhou, China). The surface morphologies, particle diameters, and surface chemical properties of the N,F-CDs were meticulously measured by transmission electron microscopy (TEM, JEM−2100, JEOL, Tokyo, Japan) and X-ray photoelectron spectroscopy (XPS, ESCALAB 250Xi, Thermo Fisher Scientific, Waltham, MA, USA), respectively. Fluorescence and ultraviolet spectra data were recorded via a fluorescence spectrophotometer (F−4700, HITACHI, Tokyo, Japan) and an ultraviolet (UV) spectrophotometer (UV1900, SHIMADZU, Tokyo, Japan), separately. Fourier transform infrared (FT-IR) spectra were acquired by a FT-IR spectrometer (Nicolet iS50, Thermo Fisher Scientific, Waltham, MA, USA).

### 2.3. Synthesis of N,F-CDs

The N,F-CDs were synthesized by referring to the method proposed by Weng’s research group with significant modifications [27]. Briefly, tetrafluoroterephthalic acid (0.9524 g) was dissolved in purified water (60 mL) in a beaker with a volume of 250 mL. Then, 20 mL of tetraethylenepentamine was added and stirred thoroughly. Subsequently, the homogeneous solution was added into a polytetrafluoroethylene reactor (250 mL) for the reaction (180 °C, 24 h). Then, the mixture was naturally cooled to room temperature and filtered (through a 0.22 μm filter membrane). After that, the solution was transferred into a dialysis bag (MW 500) for purification (48 h). Finally, the mixture was freeze-dried and prepared as a stock solution with a concentration of 3 mg/mL and stored at 4 °C for later use.

### 2.4. Fluorescence Sensing Detection

Firstly, aloin stock solution (200 μmol/L) was prepared by transferring a solution with 4.2 mg of aloin into a 50 mL volumetric flask, diluted with phosphate-buffer solution (pH 7.0, 0.1 M) to volume. Then, aloin solutions with a series of concentrations (0.1, 1, 5, 10, 15, 20, 25, 50, 70, 90, and 110 μmol/L) were prepared. Secondly, N,F-CD stock solution (3 mg/mL) was diluted to a 0.04 mg/mL solution with pure water. After that, quantitative filter paper was immersed in the N,F-CD solution (0.04 mg/mL) for 20 min and dried in darkness. Subsequently, the N,F-CD-coated test strips were cut into small pieces with a size of 2 × 3 mm; then, one of the small pieces was fixed to the center of a baseplate (0.5 cm × 5 cm) for later application; an image of the N,F-CD-coated test strips is shown in Figure S2. In the end, 1 μL of aloin solution was dropped onto the N,F-CD-coated test strips and, after drying, the fluorescence intensity was measured by a miniaturized fluorimeter.

The maximum excitation length and fluorescence sensing mechanism of the N,F-CDs for aloin were studied using a fluorescence spectrophotometer and a UV spectrophotometer. What is more, the influence of the pH was investigated by dissolving aloin into solutions with diverse pH levels (6.0, 6.5, 7.0. 7.5, and 8.0). The concentration of N,F-CDs was optimized by immersing quantitative filter paper into the N,F-CD solutions (0.01, 0.02, 0.03, 0.04, 0.05, and 0.06 mg/mL) for 20 min, and detecting the fluorescence intensity of the N,F-CD-coated test strips by a miniaturized fluorimeter.

### 2.5. Stability and Anti-Interference of N,F-CDs

The stability of the N,F-CD-coated test strips was assessed. In detail, the N,F-CD-coated test strips were placed in darkness at room temperature for 7 days, and their fluorescence intensity was detected once every day. Additionally, the stability of the N,F-CDs was tested by immersing them in NaCl solutions (0.01, 0.02, 0.03, 0.04, 0.05, and 0.06 M) for more than one hour. Meanwhile, the influence of light (72 h) and temperature (25~80 °C) on the fluorescence intensity of the N,F-CD-coated test strips was investigated.

The anti-interference of the N,F-CD-coated test strips was scrupulously evaluated. First, a solution containing one kind of compound or ion with a concentration of 80 μmol/L was prepared. Furthermore, a binary solution containing aloin and one kind of compound or ion was prepared. A total of 1 μL of the above solution was dropped onto the N,F-CD-coated test strips, and the fluorescence intensity was detected by a miniaturized fluorimeter.

### 2.6. Analysis of Aloin in Real Samples

Fetal bovine serum was diluted 30-fold with phosphate-buffer solution (pH 7.0, 0.1 M). The orange juice was purchased from Tianmao supermarket in Yantai University. The orange juice was diluted 50 times with purified water. Meanwhile, 20 µL of urine was mixed with 60 µL of methanol, and centrifuged to remove protein. After that, the mixture was dried by N_2_, and 50 µL of purified water was added [28]. Subsequently, the diluted fetal bovine serum, orange juice, and urine samples were spiked with specific concentrations of aloin (0, 5, 15, and 30 μmol/L) to evaluate the N,F-CD-coated test strips, and the results were verified by a UV spectrophotometer.

## 3. Results and Discussion

### 3.1. Characterization of N,F-CDs

**Figure 2:**
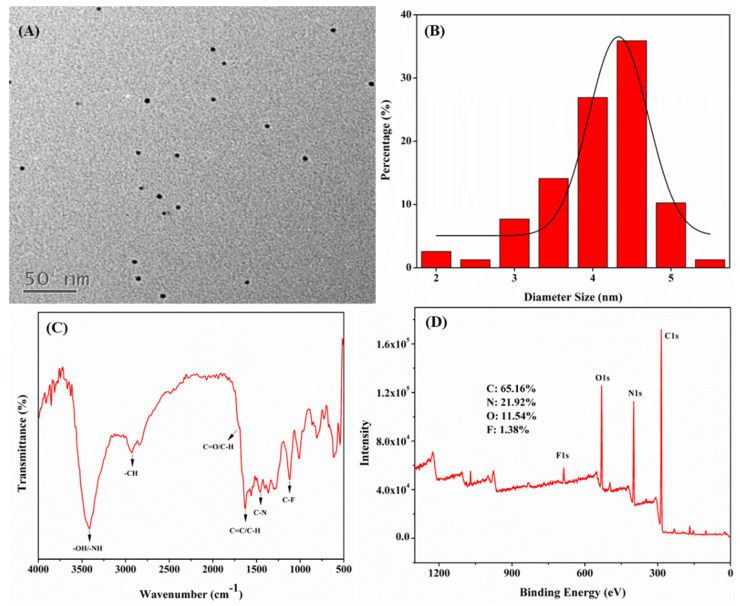
(**A**) TEM image, (**B**) histogram of particle size distribution, (**C**) FT-IR spectrum, and (**D**) XPS spectrum of N,F-CDs, respectively.

As illustrated in Figure 2A,B, the as-prepared N,F-CDs possessed good spherical morphology and good dispersion; their particle sizes ranged from 2 to 5.5 nm, with an average particle size of 4.3 nm. The FT-IR analysis (Figure 2C) suggested that some functional groups could be measured on the N,F-CDs. The absorption peaks near 3415, 2927, 1699, and 1631 cm^−1^ were caused by the stretching vibrations of -OH/-NH, -OH, C-H, and C=O, respectively [29,30]. The peaks at 1457 cm^−1^ and 1120 cm^−1^ were attributed to the stretching vibrations of C-N and C-F, separately [27]. XPS data is shown in Figure 2D; four typical peaks of C1s (284.81 eV), N1s (398.42 eV), O1s (530.43 eV), and F1s (686.47 eV) were observed, and the atomic contents of C, N, O, and F were 65.16%, 21.92%, 11.54%, and 1.38%, respectively. Furthermore, as shown in Figure S3A–D, the high-resolution spectra of C1s (284.8 eV for C-C, 285.45 eV for C-N/C-O, and 287.35 eV for C=O), N1s (398.6 eV for N-H, 399.7 eV for C-N and 401 eV for graphitic nitrogen), O1s (530.45 eV for C-O and 531.7 eV for C=O), and F1s (686.75 eV for C-F) further confirmed the successful preparation of N,F-CDs, and were consistent with those of the FT-IR [27,31,32,33].

### 3.2. Fluorescence Mechanism

**Figure 3:**
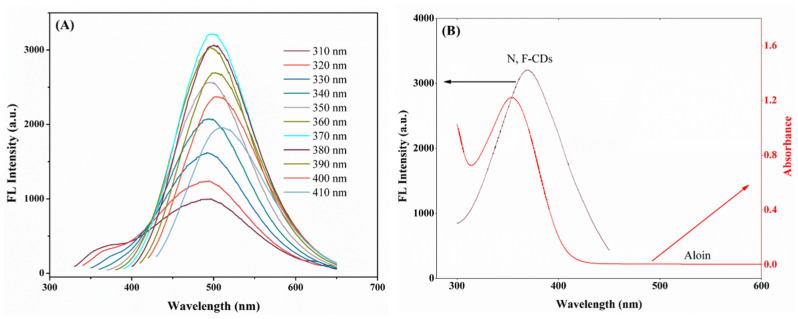
(**A**) Fluorescence emission spectra of N,F-CDs excited with excitation wavelength of 310~410 nm. (**B**) UV-vis absorption spectra of aloin and fluorescence excitation spectrum of N,F-CDs.

Additionally, the fluorescence emission spectra of the as-prepared N,F-CDs solution are shown in Figure 3A; from 320 nm to 370 nm, the fluorescence intensity increased obviously, and decreased gradually after 370 nm. Furthermore, with the excitation wavelength increased from 310 nm to 410 nm, an obvious red-shifting of the maximum emission wavelength could be observed (from 495 nm to 510 nm). The above results were mainly caused by the energy level and the highly sp^2^-hybridized atomic domain properties of the N,F-CDs [34]. In addition, the maximum emission wavelength near 500 nm could be acquired under an excitation wavelength of 370 nm, which revealed that the miniaturized fluorimeter (excitation wavelength: 365 nm, emission wavelength: 500 nm) was suitable for this work.

Moreover, as shown in Figure S4, with the increase of the aloin concentration (0.1~110 μmol/L), the fluorescence intensity of the N,F-CD-coated test strips decreased obviously, which demonstrated the possible quenching mechanism of the inner filter effect (IFE), and of static and dynamic quenching [35,36,37,38]. In Figure 3B, the UV-vis absorption spectra of aloin could be largely overlapped by the fluorescence excitation spectrum of the N,F-CDs, which implied that the primary IFE occurred. Then, the relationship between the suppressed efficiency (%E) and concentration of aloin was studied. In Figure S5A,B, the main contributor was the IFE. Additionally, a Stern–Volmer study was performed to investigate the independence pattern of K_SV_ (quenching constant) at diverse temperatures (such as 313, 323, and 333K), which verified that the IFE is the only contributor. The UV-vis spectra of N-CDs with and without aloin are shown in Figure S6A; these very similar spectra excluded the static mechanism. In the end, as exhibited in Figure S6B, the fluorescence lifetimes of N,F-CDs without and with aloin were 4.25 ns and 4.32 ns; this inconspicuous change indicates that no fluorescence resonance energy transfer (FRET) and dynamic quenching effect existed between the N,F-CDs and the aloin [27]. In brief, static and dynamic quenching effects were ruled out, and only the IFE existed in the quenching process [39].

### 3.3. Optimization of Determination Conditions

**Figure 4:**
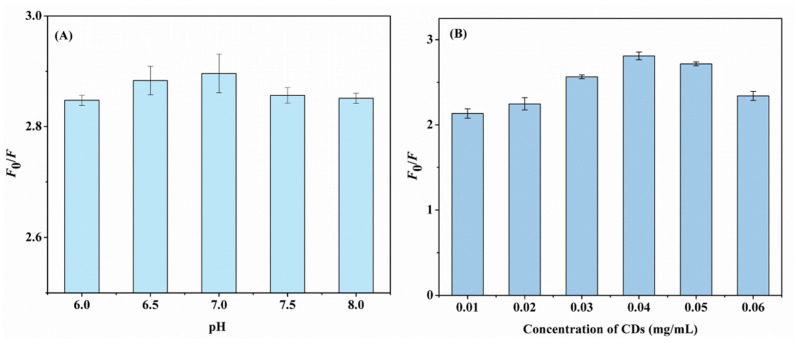
(**A**) The effect of aloin solution pH on the fluorescence intensity of N,F-CD-coated test strips. (**B**) The fluorescence response of the test papers with different concentration of N,F-CDs.

The pH and concentration of the N,F-CDs were optimized to enhance the efficacy of aloin detection. The parameter quenching efficiency (*F*_0_/*F*) was used to evaluate the fluorescence property of the system. In this equation, *F*_0_/*F* = 1 + *K*_SV_*[C]*, where *F* and *F*_0_ are the fluorescence intensities of N,F-CD-coated test strips with and without aloin, and *K*_SV_ and *[C]* are the quenching constant and actual concentration of aloin, separately. As exhibited in Figure 4A, the pH range of 6.0~8.0 was used to optimize the quenching efficiency of the N,F-CD-coated test strips. In addition, the fluorescence intensity of N,F-CDs under different pH conditions is presented in Figure S7, and the results were consistent with those of Figure 4A. Finally, the optimal pH 7 was utilized in the following experiment. A possible reason is that the sensing performance between the aloin and the N,F-CDs could be influenced by the pH of the solution.

Meanwhile, the sensing performance of the system could be greatly influenced by the concentration of CDs. Then, the concentration of N,F-CDs within 0.01~0.06 mg/mL was optimized. The *F*_0_/*F* increased as the concentration of N,F-CDs increased from 0.01 to 0.04 mg/mL, and decreased gradually after that, which revealed that the optimal value was 0.04. The next experiment was carried out using 0.04 mg/mL of N,F-CDs.

### 3.4. Linearity and Sensitivity of N,F-CD-Coated Test Strips

**Figure 5:**
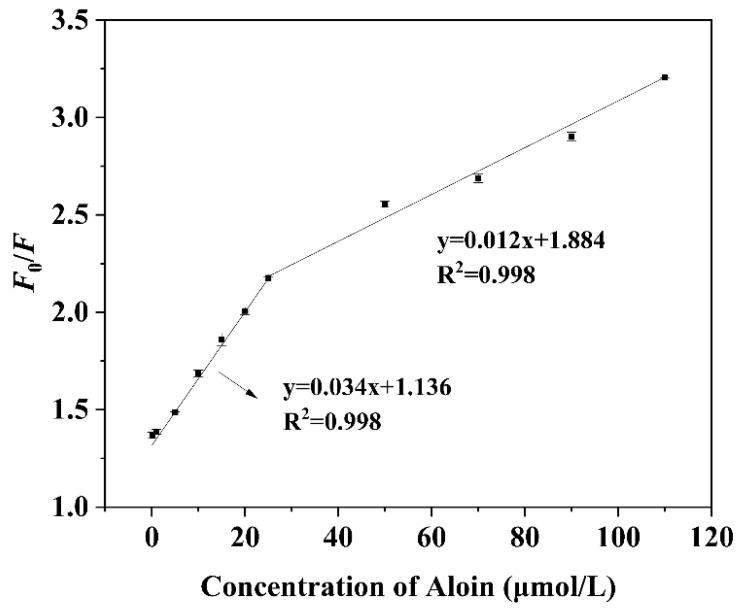
The linear relationship between *F*_0_/*F* and a certain concentration of aloin over the ranges of 0.1 to 25 μM and 25 to 110 μM.

The as-constructed N,F-CD-coated test strips were supposed to be efficient at the fluorescence detection of aloin, due to the unique optical performance of N, F-CDs. As illustrated in Figure 5, an excellent linear relationship between *F*_0_/*F* and the concentration of aloin could be acquired. In the range of 0.1~25 μM, the equation is y = 0.034x + 1.136 (R^2^ = 0.998); while in the range of 25~110 μM, the equation is y = 0.012x + 1.884 (R^2^ = 0.998). Through the 3σ/slope principle, the limit of detection (LOD) of aloin was calculated as 31.8 nM (3σ/k, n = 3), which was lower than previously proposed fluorescence methods (0.052 μM) [40]. The above results further demonstrate that the N,F-CD-coated test strips are suitable for aloin detection.

### 3.5. Selectivity, Stability, and Reproducibility

**Figure 6:**
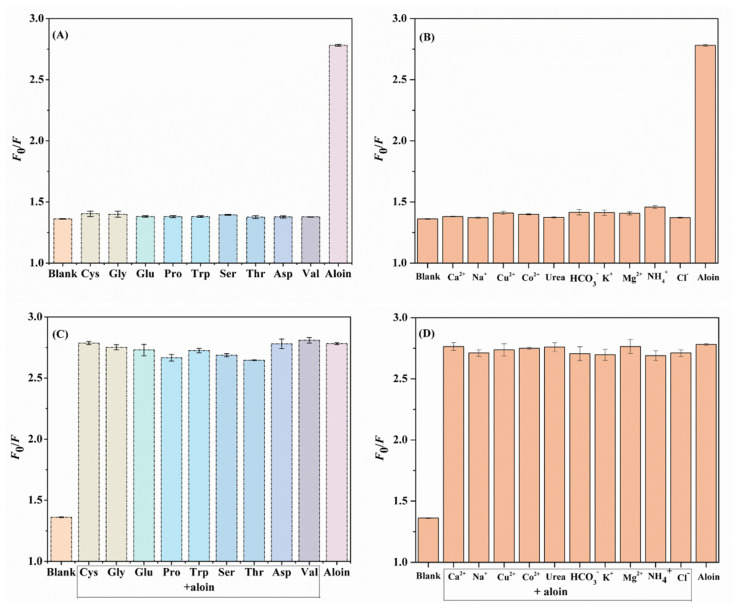
(**A**,**B**) The effect of interfering substances on the fluorescence intensity of N,F-CDs without aloin. (**C**,**D**) The effect of interfering substances on the fluorescence intensity of N,F-CDs in the presence of aloin.

The analytical performances (including selectivity and stability) of the N,F-CD-coated test strips toward targets were crucial. It is universally known that diverse biological interference substances are existent in the system, thus, research on selectivity is very valuable. As displayed in Figure 6A–D, typical amino acids (Cys, Gly, Glu, Pro, Trp, Ser, Thr, Asp, and Val), common metal cations (including Na^+^, K^+^, Ca^2+^, NH_4_^+^, Mg^2+^, Cu^2+^, Co^2+^, HCO_3_^−^, and Cl^−^), and urea were used to evaluate the selectivity of the N,F-CD-coated test strips [41]. The chemical structural formulas of various amino acids can be seen in Figure S8. The *F*_0_/*F* values of solutions containing aloin were much higher than others without aloin; the possible reason being that the interactions between the N,F-CDs and the aloin were different from those for other analytes. The above results further confirmed that the N,F-CD-coated test strips possessed excellent selectivity and anti-interference ability towards aloin.

**Figure 7:**
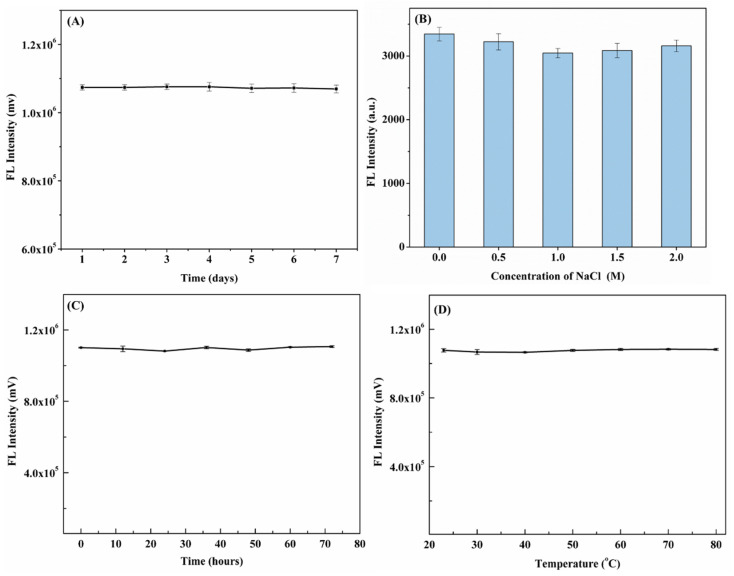
(**A**) The stability of N,F-CD-coated test strips after 7 days; (**B**) the effect of NaCl concentration on the fluorescence intensity of N,F-CDs; (**C**) the influence of light treatment on the fluorescence intensity of N,F-CD-coated test strips after 72 h; (**D**) the stability of N,F-CD-coated test strips at different temperatures (25~80 °C).

The stability of the N,F-CDs in darkness for 7 days was investigated. Moreover, as revealed in Figure 7A,B, for N,F-CD-coated test strips, nearly no obvious fluorescence intensity decline could be observed in 7 days. Otherwise, the N,F-CDs were immersed in a NaCl solution for about one hour, and nearly no distinct fluorescence intensity change could be found. Moreover, as depicted in Figure 7C,D, the N,F-CD-coated test strips showed excellent light stability (rate of change of fluorescence intensity, −0.53%~1.76%) and temperature stability (rate of change of fluorescence intensity, −0.59%~1.06%). In addition, the fluorescence intensity of the N,F-CD-coated test strips was tested in an independent laboratory. The average fluorescence intensity and RSD values of the N,F-CD-coated test strips in the A803 laboratory and B406 laboratory were 1,101,082 mv and 1,096,605 mv, and 0.19% and 0.90%, respectively, which indicated this method had good reproducibility. The results demonstrate that the N,F-CDs and N,F-CD-coated test strips had wonderful selectivity, stability, and reproducibility, and that the resulting system is suitable for the quantitative detection of aloin.

### 3.6. Analysis of Aloin in Bovine Serum Samples

Bovine serum is commonly used as a matrix to evaluate the properties of a fluorescent probe for the measurement of compounds (aloin, biothiols, etc.) harmful to human health [40,42]. The possible reasons are that the bovine serum is easily available and possesses similar components to human serum. Furthermore, the content of aloin in orange juice and urine was investigated. The practicability of the N,F-CD-coated test strips was assessed by detecting aloin in orange juice, urine, and bovine serum samples. As exhibited in Table 1, the recovery of the aloin in these samples ranged from 96.9 to 106.1%, with a relative standard deviation (RSD) of no more than 3.9%. Additionally, the fluorescence method was verified by the frequently used UV approach. As can be seen in Figure S9, the equation of the UV method for aloin detection is y = 0.01x + 0.003 (R^2^ = 0.9996, 0.5~38 M). The ranges of recovery and RSD of the UV method were 94.7~104.9% and 0.2~2.4%, respectively. In this investigation, the recovery results for fluorescence method were slightly superior to those of the UV method, which demonstrates that the proposed fluorescence method is suitable for aloin determination.

As illustrated in Table S1 [9,40,43,44], diverse methods for the determination of aloin are presented; the results demonstrate that our fluorescence method possesses good properties (involving high sensitivity and recovery, a wide linear range, good repeatability, and high speed), which indicates that the results of the N,F-CD-coated test strips were not obviously different from other methods (HPLC, capillary electrophoresis, etc.). Furthermore, as shown in Table S2 [45,46,47,48], the performances (such as sensitivity and linear range) of the N,F-CD-coated test strips were superior to other CD-based strip sensors for the detection of phenolic compounds, which further verifies that the N,F-CD-coated test strips are suitable for aloin determination.

## 4. Conclusions

In this investigation, a fluorescence sensor using N,F-CDs coupled with a miniaturized fluorimeter was successfully constructed, and aloin in real serum samples was successfully detected via this system. The as-synthesized N,F-CDs possessed a large Stokes shift and could emit a highly stable green fluorescence. The fluorescence of the N,F-CDs could be effectively quenched by aloin through the inner filter effect. Furthermore, the linear relationship between *F*_0_/*F* and aloin concentration was established in the ranges of 0.1~25 μM and 25~110 μM, and the LOD was 31.8 nM. Meanwhile, this fluorescent sensor system had satisfactory recovery and excellent RSD in the actual sample detection. Therefore, N,F-CD-coated test strips will arouse wide interest in the convenient fluorescence sensing of aloin in diverse real samples.

## Figures and Tables

**Table 1:** Real samples spiked with aloin estimated by the fluorescence method and the UV method.

Methods	Samples	Spiked (µM, n = 3)	Measured Signals (n = 3)	Detected (µM, n = 3)	RSD (%, n = 3)	Recovery (%)
Fluorescence method	Bovine serum	0	828,971.50	NA	--	--
		5	718,723.50	5.10	2.8	102.0
		15	584,154.50	15.91	0.7	106.1
		30	477,703.47	29.08	3.2	96.9
	Orange juice	0	824,364.00	NA	--	--
		5	733,779.33	5.03	3.9	100.6
		15	586,502.33	15.15	3.7	101.0
		30	477,792.00	29.96	3.4	99.9
	Urine	0	846,196.67	NA	--	--
		5	729,600.33	4.91	1.7	98.2
		15	577,194.67	15.88	2.8	105.9
		30	481,820.33	29.84	3.8	99.5
UV method	Bovine serum	0	0	NA	--	--
		5	0.05	4.73	2.4	94.7
		15	0.15	14.47	1.1	96.4
		30	0.30	29.63	0.7	98.8
	Orange juice	0	0	NA	--	--
		5	0.04	4.54	1.4	98.8
		15	0.14	15.15	0.7	101.0
		30	0.28	30.96	0.3	103.2
	Urine	0	0	NA	--	--
		5	0.04	4.25	1.4	85.0
		15	0.15	15.74	0.8	104.9
		30	0.28	30.17	0.2	100.6

NA not applicable.

## Data Availability

The data will be made available on request.

## Supplementary Materials

The following supporting information can be downloaded at: https://www.mdpi.com/article/10.3390/biom15071052/s1, Figure S1: Chemical structure of aloin. Figure S2: Image of the N,F-CDs coated test strips. Figure S3: High-resolution survey of (A) C1s, (B) N1s, (C) O1s and (D) F1s of N,F-CDs. Figure S4: (A–C) Fluorescence spectra of N,F-CDs in presence of aloin ((A) 0.1 μM, (B) 30 μM, (C) 110 μM). Figure S5: (A,B) %E for corrected and observed fluorescence of N,F-CD-coated test strips after adding increasing concentrations of aloin (n = 3). (C,D) Stern-Volmer plots of N,F-CD-coated test strips+ aloin system at different temperatures (n = 3). Figure S6: UV-vis absorption spectra (A) and fluorescence decay curves (B) of N,F-CDs with and without aloin. Figure S7: Fluorescence intensity of N,F-CDs solution with different pH. Figure S8: The chemical structural formulas of various amino acids. Figure S9: Standard curve for detecting aloin through UV method. Table S1: Comparison of diverse strategies for determination of aloin. Table S2: Comparison of other CDs-based strip sensors for analysis of phenolic compound. References [43,44,45,46,47,48] are cited in the supplementary materials.
