# Supplementary material for: Sensitive and Facile Detection of Aloin via N,F-CD-Coated Test Strips Coupled with a Miniaturized Fluorimeter
Source: Biomolecules. 2025 Jul 21;15(7):1052. doi: 10.3390/biom15071052 (PMC12294048; doi:10.3390/biom15071052)
Supplement: Supplementary file 1 [file biomolecules-15-01052-s001.zip › biomolecules-3695020-supplementary.pdf]

Supporting Information

Sensitive and Facile Detection of Aloin via N,F-CD-Coated Test Strips Coupled with a Miniaturized Fluorimeter

Guo Wei [1,†], Chuanliang Wang [2,3,†], Rui Wang [1], Peng Zhang [1], Xuhui Geng [2], Jinhua Li [4], Abbas Ostovan [4], Lingxin Chen [4] and Zhihua Song [1,*]

[1] School of Pharmacy, Collaborative Innovation Center of Advanced Drug Delivery System and Biotech Drugs in Universities of Shandong, Key Laboratory of Molecular Pharmacology and Drug Evaluation (Yantai University), Ministry of Education, Yantai University, Yantai 264005, China
[2] CAS Key Laboratory of Separation Sciences for Analytical Chemistry, Dalian Institute of Chemical Physics, Chinese Academy of Sciences, 457 Zhongshan Road, Dalian 116023, China
[3] University of Chinese Academy of Sciences, Beijing 100049, China
[4] CAS Key Laboratory of Coastal Environmental Processes and Ecological Remediation, Research Center for Coastal Environmental Engineering and Technology, Yantai Institute of Coastal Zone Research, Chinese Academy of Sciences, Yantai 264003, China
* Correspondence: zhihuasong08@yeah.net; Tel./Fax: +86-535-6706-066
† These authors contributed equally to this work.

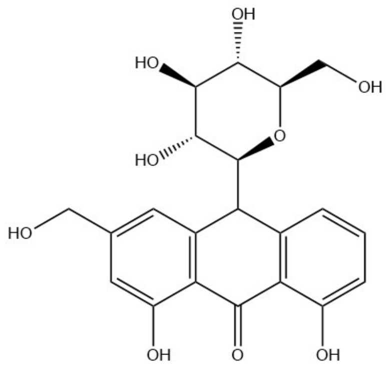

Figure S1 Chemical structure of aloin.

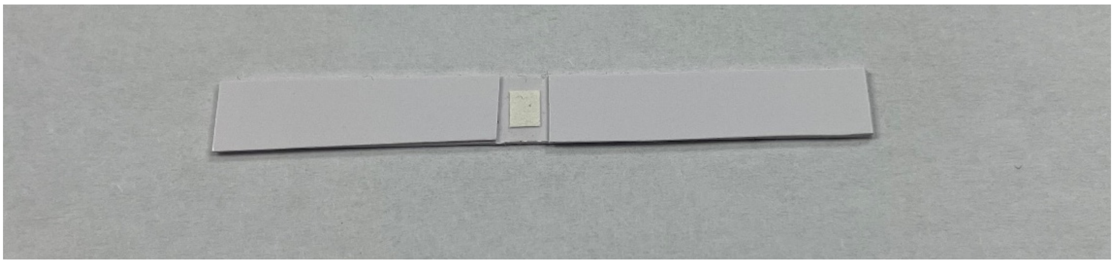

Figure S2 Image of the N,F-CDs coated test strips.

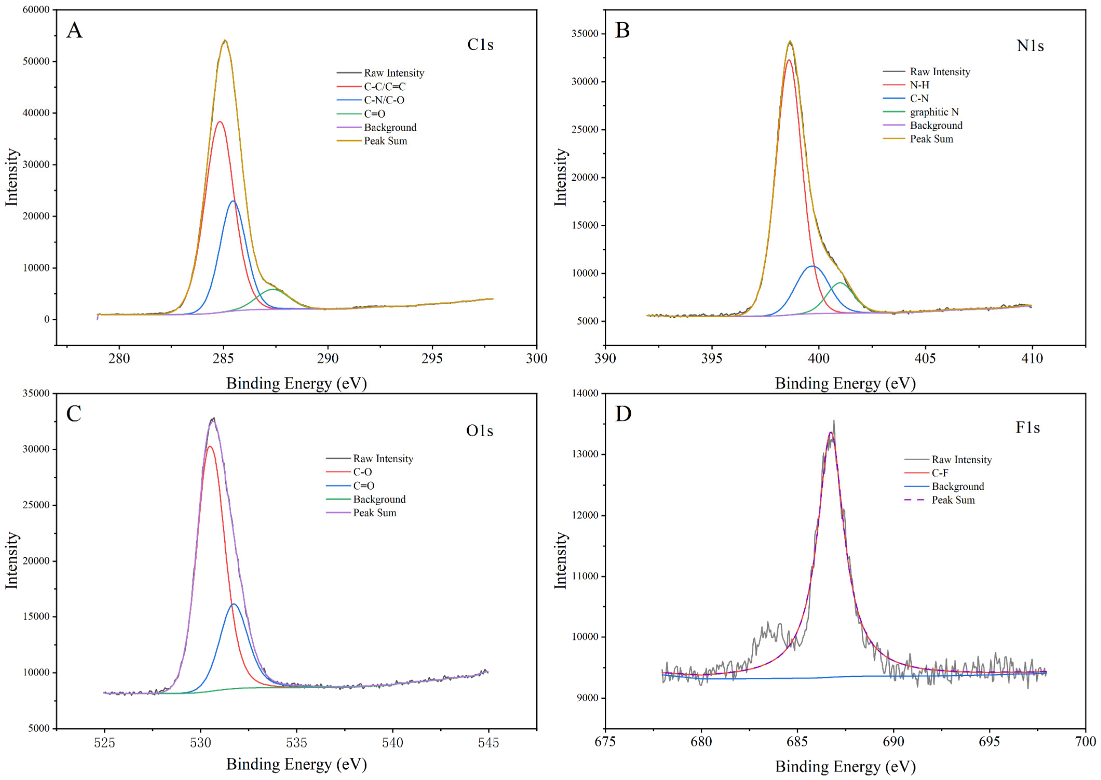

Figure S3 High-resolution survey of (A) C1s, (B) N1s, (C) O1s and (D) F1s of N,F-CDs.

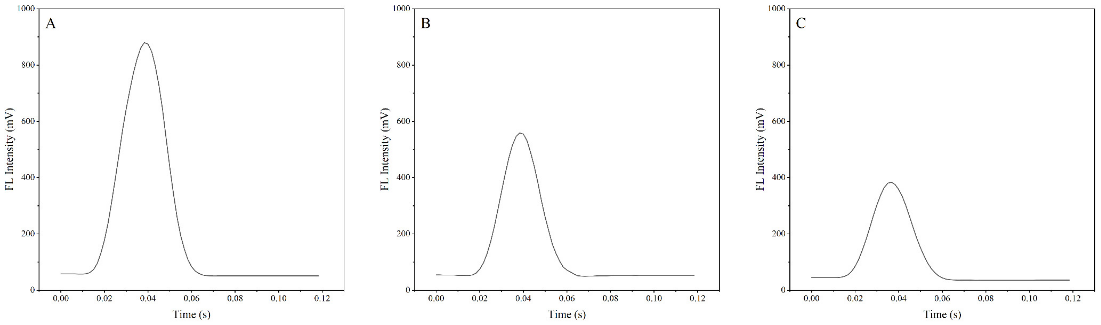

Figure S4 (A-C) Fluorescence spectra of N,F-CDs in presence of aloin ((A) 0.1 μM, (B) 30 μM, (C) 110 μM).

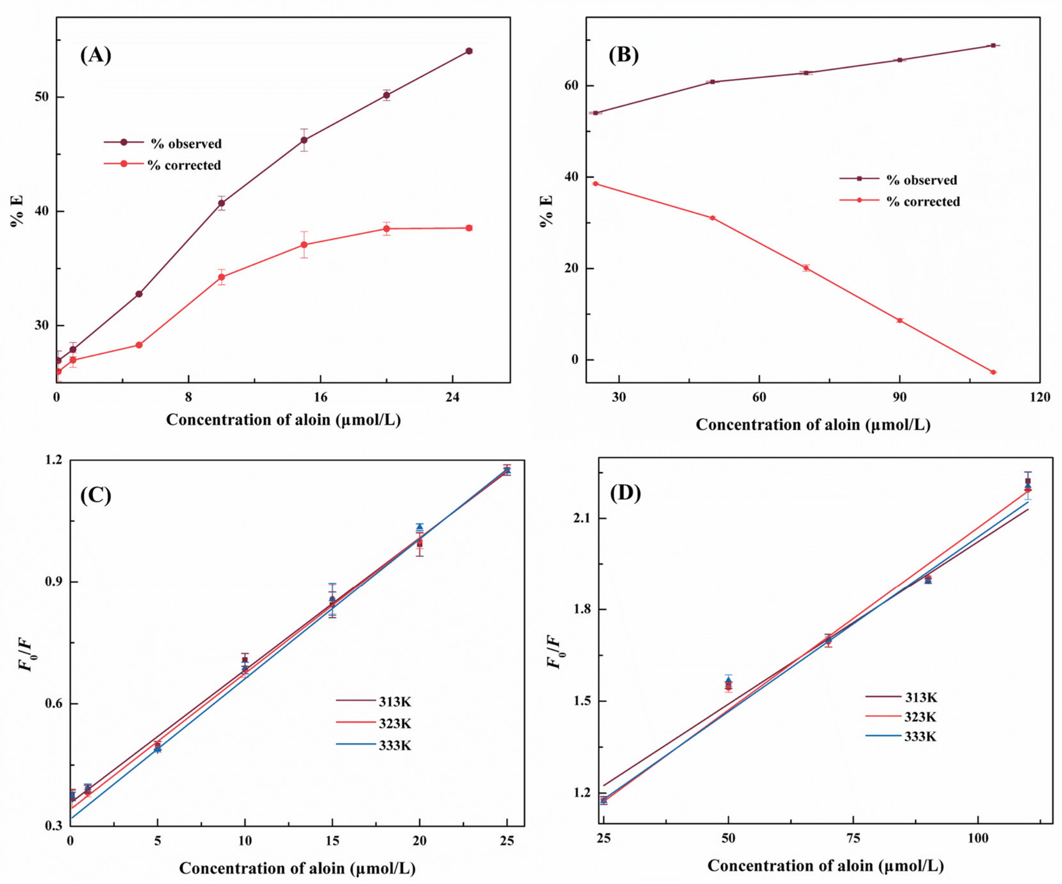

Figure S5 (A&B) %E for corrected and observed fluorescence of N,F-CD-coated test strips after adding increasing concentrations of aloin (n = 3). (C&D) Stern-Volmer plots of N,F-CD-coated test strips+ aloin system at different temperatures (n = 3).

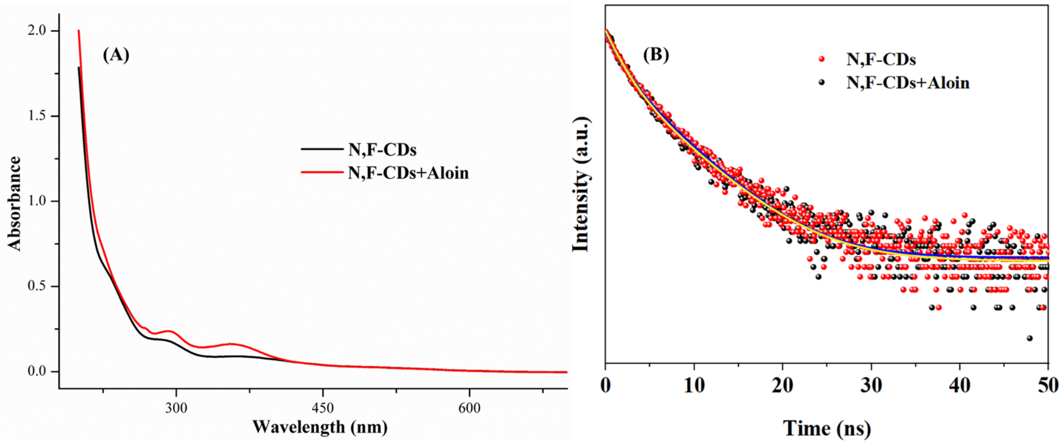

Figure S6 UV-vis absorption spectra (A) and fluorescence decay curves (B) of N,F-CDs with and without aloin.

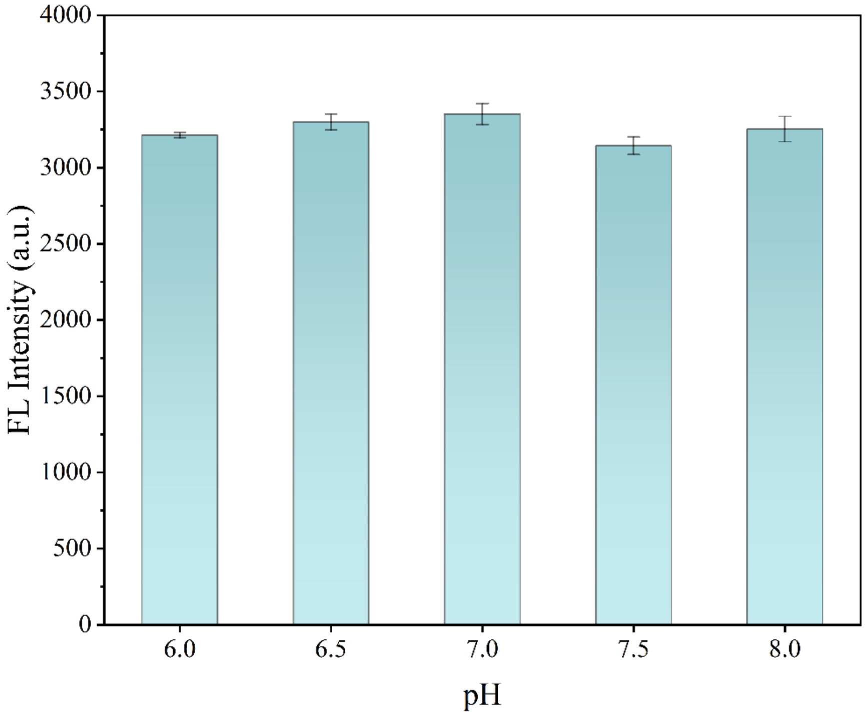

Figure S7 Fluorescence intensity of N,F-CDs solution with different pH.

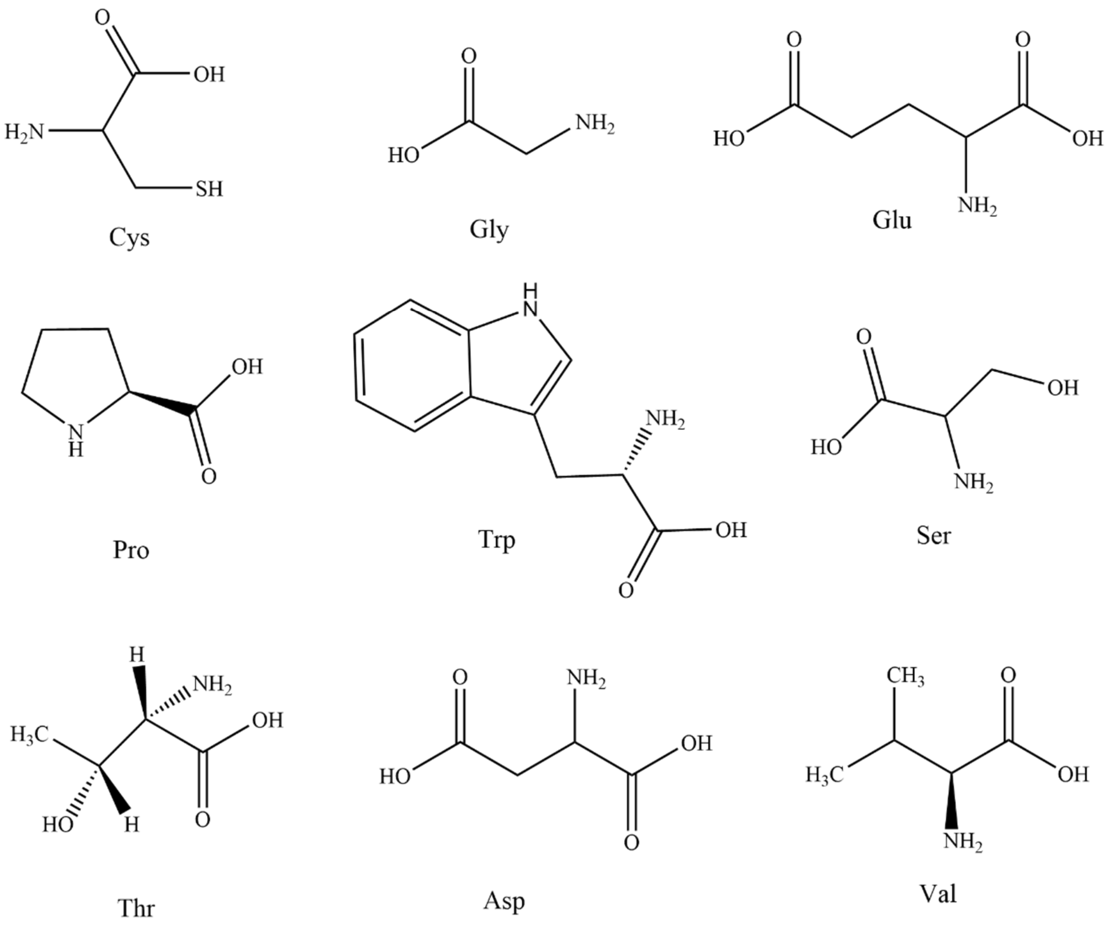

Figure S8 The chemical structural formulas of various amino acids.

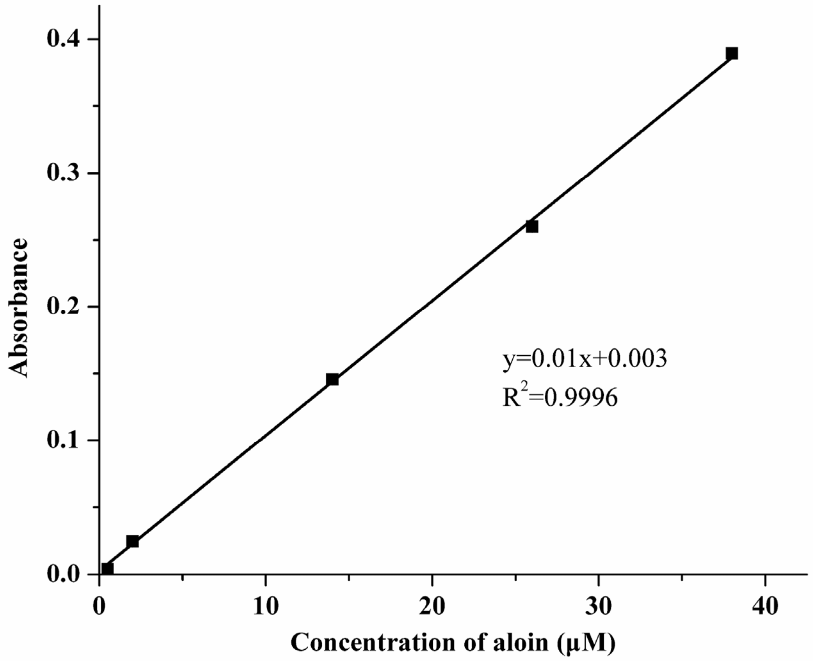

Figure S9 Standard curve for detecting aloin through UV method.

Table S1 Comparison of diverse strategies for determination of aloin.

Method	Linear range (μmol/L)	LOD (μmol/L)	Samples	Recovery (%)	RSD (%)	Time (min)	References
HPLC	388.39~8489.13<br>99.67~5867.20	--	Latex<br>Gel	--	0.81~4.42	24	[1]
HPLC	0.136~1362.36<br>0.103~1027.74	0.041<br>0.031	Shampoo, Soap, Hair Conditioner, Treatment oil, Powder	98.5~101.6<br>98.0~101.3	0.1~1.8	13	[2]
Capillary electrophoresis	0.0597~1.195<br>0.0597~1.195	0.0175<br>0.0179	Aloe preparations<br>Aloe functional food	97~109.1<br>92.8~106.2	1.8~5.1<br>3.2~3.9	0.67	[3]
Fluorescence determination	0.5-200	0.052	Bovine serum<br>Black tea	90.8~106.8	2.13~3.44	1	[4]
Fluorescence determination	0.1~25<br>25~110	0.0318	Bovine serum	96.9~106.1	0.7~3.2	5	This work

Table S2 Comparison of other CDs-based strip sensors for analysis of phenolic compound.

Material	Analyte	Analysis method	Linear range (μmol/L)	LOD (μmol/L)	Samples	References
Y-CDs, G-CDs, B-CDs	Epinephrine (Ep),<br>Norepinephrine (NE),<br>Levodopa (L-DOPA)	Qualitative analysis	Ep: 1~100<br>NE: 0~100<br>L-DOPA: 0~500	Ep: 7.2<br>NE: 2.6<br>L-DOPA: 19.1	Human Serum	[5]
ZnO@ZIF-8/CDs@MIPs	Bisphenol A	Qualitative analysis	--	--	Canned fish	[6]
CDs@Eu-AMP	Bisphenol A	Semi quantitative detection	0, 20, 40, 60, 80, 100	--	Environmental water	[7]
DNA-CuNC/CDs	Acetaminophen	Semi quantitative detection	0, 10, 30, 70	--	Human<br>serum samples	[8]
N, F-CDs	Aloin	Qualitative analysis<br>Quantitative analysis	0.1~25<br>25~110	0.0318	Bovine serum, orange juice<br>urine	This work

Reference

1. Sánchez-Machado, D.I.; López-Cervantes, J.; Mariscal-Domínguez, M.F.; Cruz-Flores, P.; Campas-Baypoli, O.N.; Cantú-Soto, E.U.; Sanches-Silva, A. An HPLC procedure for the quantification of aloin in latex and gel from *Aloe barbadensis leaves*. *J. Chromatogr. Sci.* **2017**, *55(3)*, 251-257.
2. Sibhat, G.; Kahsay, G.; Van Schepdael, A.; Adams, E. Evaluation of aloins, pH and moisture in aloe leaf gel-based personal care products. *Int. J. Cosmet. Sci.* **2022**, *44(1)*, 74-81.
3. Xiao, M.W.; Bai, X.L.; Liu, Y.M.; Yang, L.; Hu, Y.D.; Liao. X. Rapid quantification of aloin A and B in aloe plants and aloe-containing beverages, and pharmaceutical preparations by microchip capillary electrophoresis with laser induced fluorescence detection. *J. Sep. Sci.* **2018**, *41(19)*, 3772-3781.
4. Cai, Z.F.; Deng, C.H.; Wang, J.; Zuo, Y.; Wu, J.L.; Wang, X.P.; Lv, T.Z.; Wang, Y.Y.; Feng, D.Y.; Zhao, J.; Zhang, C.F.; Zhang, J.M. Sensitive and selective determination of aloin with highly stable histidine-capped silver nanoclusters based on the inner filter effect. *Colloid. Surface. A* **2021**, *627*, 127224.
5. Zhang, J.J.; An, J.; Han, Y.Q.; Fang, J.A.; Liu, Y.F. Spermine induced in-situ synthesis of polychromatic carbon nanodots towards smartphone-readable ratiometric fluorescence sensing of multiple catecholamines. *Talanta* **2025**, *285*, 127292.
6. Liu, X.Q.; Wang, T.; Wang, Y.Q. Selective and ratiometric fluorescence sensing of bisphenol A in canned food based on portable fluorescent test strips. *Anal. Chim. Acta* **2023**, *1240*, 340728.
7. Li, Y.X.; Min, Q.; Wang, Y.F.; Zhuang, X.M.; Hao, X.W; Tian, C.Y.; Fu, X.L.; Luan, F. A portable visual coffee ring based on carbon dot sensitized lanthanide complex coordination to detect bisphenol A in water. *RSC Adv.* **2022**, *12(12)*, 7306-7312.
8. Bu, X.N.; Fu, Y.X.; Jiang, X.W.; Jin, H.; Gui, R.J. Self-assembly of DNA-templated copper nanoclusters and carbon dots for ratiometric fluorometric and visual determination of arginine and acetaminophen with a logic-gate operation. *Microchim. Acta* **2020**, *187(3)*, 154.
